# The Regulated Secretory Pathway and Human Disease: Insights from Gene Variants and Single Nucleotide Polymorphisms

**DOI:** 10.3389/fendo.2013.00096

**Published:** 2013-08-06

**Authors:** Wei-Jye Lin, Stephen R. Salton

**Affiliations:** ^1^Department of Neuroscience, Icahn School of Medicine at Mount Sinai, New York, NY, USA; ^2^Friedman Brain Institute, Icahn School of Medicine at Mount Sinai, New York, NY, USA; ^3^Department of Geriatrics, Icahn School of Medicine at Mount Sinai, New York, NY, USA

**Keywords:** single nucleotide polymorphism (SNP), dense core granule (DCG), insulin, brain-derived neurotrophic factor (BDNF), chromogranin, neurotrophin, proopiomelanocortin (POMC), prohormone convertase

## Abstract

The regulated secretory pathway provides critical control of peptide, growth factor, and hormone release from neuroendocrine and endocrine cells, and neurons, maintaining physiological homeostasis. Propeptides and prohormones are packaged into dense core granules (DCGs), where they frequently undergo tissue-specific processing as the DCG matures. Proteins of the granin family are DCG components, and although their function is not fully understood, data suggest they are involved in DCG formation and regulated protein/peptide secretion, in addition to their role as precursors of bioactive peptides. Association of gene variation, including single nucleotide polymorphisms (SNPs), with neuropsychiatric, endocrine, and metabolic diseases, has implicated specific secreted proteins and peptides in disease pathogenesis. For example, a SNP at position 196 (G/A) of the human brain-derived neurotrophic factor gene dysregulates protein processing and secretion and leads to cognitive impairment. This suggests more generally that variants identified in genes encoding secreted growth factors, peptides, hormones, and proteins involved in DCG biogenesis, protein processing, and the secretory apparatus, could provide insight into the process of regulated secretion as well as disorders that result when it is impaired.

## General Overview of Regulated Secretion

Neuronal and endocrine peptides, growth factors, and hormones maintain physiological homeostasis. Their release is therefore tightly controlled by regulated secretion (1). Messenger RNAs (mRNAs) are translated and secreted proteins enter the cisternae of the rough endoplasmic reticulum (RER), are transported to the trans-Golgi network (TGN), are targeted into immature dense core granules (DCGs), and are retained in mature DCGs (also called large dense core vesicles or LDCVs), in endocrine and neuronal cells. Proteins including members of the granin family, such as chromogranin A (CgA), chromogranin B (CgB), secretogranin II (SgII), and secretogranin III (SgIII), the exopeptidase carboxypeptidase E (CPE), and prohormone convertases 1/3 and 2 (PC1/3 and PC2), are also sorted into DCGs, which in addition, increase the diversity of biologically active peptides through processing of granin precursor proteins (2).

Although the sorting of proteins into DCGs is not fully understood, interaction of DCG cargo proteins with TGN lipid raft-anchoring or membrane-spanning proteins (SgIII, CPE, sortilin) plays an essential role in docking and concentrating of DCG components and is critical for the correct sorting of cargo proteins and for vesicle biogenesis (3, 4). DCG sorting domains have been identified in prohormones, propeptides, and granins, and although not highly conserved, these polypeptide motifs are required for regulated secretion. After budding from the TGN, immature DCGs undergo acidification, mediated by the DCG membrane-spanning proton pump, leading to activation of resident prohormone convertases and carboxypeptidases. All granin members and most other propeptides, prohormones, and prohormone convertases are known to undergo proteolytic processing during granule maturation, which is essential for enzymatic activation and the generation of biologically active peptides.

Granin family members, including CgA, CgB, SgII, and SgIII, are the most abundant DCG components, with CgA, for example, constituting almost half of the soluble protein content of the adrenal chromaffin cell secretory granule (5). The importance of granin proteins in vesicle biogenesis has been demonstrated by overexpression of CgA or CgB in cells that lack a classical regulated secretory pathway, such as fibroblasts, which results in the production of granule-like structures and the regulated release of vesicle contents (6, 7). Moreover, gene silencing studies demonstrate reciprocal effects on DCG biogenesis, with decreased DCG number reported in PC12 cells that contain reduced levels of CgA or SgII, and in the adrenal medulla of CgA knockout mouse (8–10). Other CgA and CgB knockout mouse models, although showing no change in DCG number in adrenal medulla and other endocrine tissues, were found to have increased levels of other granin proteins, which is likely due to compensatory mechanisms (11, 12).

## Mechanisms by Which Genetic Variants Can Impact Secreted Proteins

**Figure 1:**
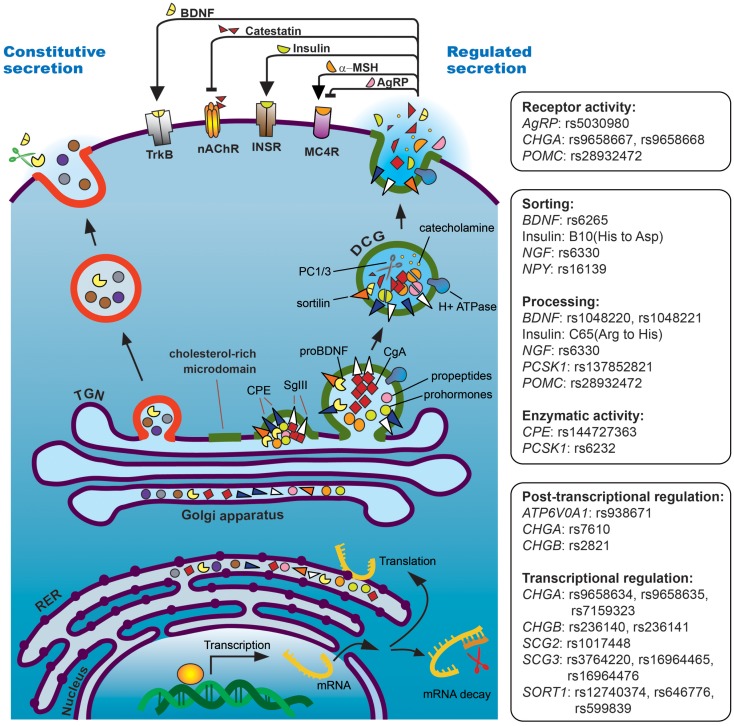
**Regulated secretory pathway of a model neuroendocrine cell**. Single nucleotide polymorphisms (SNPs) can affect granule biogenesis and release through these mechanisms: (1) SNPs in the promoter or 3′UTR of granule-associated genes modulate gene expression levels, resulting in altered granule biogenesis, (2) SNPs in the pro-domain of peptides or hormones block their processing and/or sorting into the regulated pathway, (3) SNPs in convertase/peptidase catalytic or regulatory domains reduce enzymatic activity and lead to aberrant propeptide processing, (4) SNPs in the signal peptide and/or sorting domain(s) disrupt regulated secretion, and (5) SNPs that change the processing or sequence of neuropeptides modulate membrane receptor binding affinity. TrkB, tropomyosin-related kinase B receptor; INSR, insulin receptor.

Single nucleotide polymorphisms (SNPs) are the most common genetic variations in genomic DNA, occurring 1/1200 bp on average (13). Individual SNPs submitted to the Single Nucleotide Polymorphism Database (dbSNP), a service provided by the National Center for Biotechnology Information (NCBI), are assigned a unique reference SNP ID number (rs#) to map the SNP to other external databases. Many SNPs are silent variations and their occurrence does not affect gene expression or protein function. However, a single nucleotide change has the potential to modulate protein level or function if the SNP is located: (1) in the promoter region, which may alter transcriptional activity, (2) in intronic regions, which may interfere with splicing efficiency if the SNP is found at exon-intron boundaries in splice donor sites, regulatory sequences, or splice acceptor sites, (3) in the 3′ untranslated region (3′UTR), which may affect the expression levels of either mRNA or protein due to altered *cis*-elements including mRNA destabilization sequences, AU-rich elements, and translational repressor binding sites, including miRNA targeting sites, and (4) in the protein coding region, which can cause missense or nonsense mutations. Nonsense mutations can result in premature termination of translation and the production of truncated proteins, while missense mutations can lead to loss-of-function or gain-of-function. With respect to proteins in the regulated secretory pathway, missense SNPs could impact enzymatic catalytic activity, alter peptide affinity for receptors, impair protein sorting when found in targeting motif(s) of secretory proteins, or block peptide processing when located in the cleavage sites of prohormones or propeptides (Figure 1).

## Reported SNPs in Genes Encoding Secreted Proteins that are Associated with Neuropsychiatric or Endocrine/Metabolic Disease

Neurotrophic growth factors, hormones, peptide precursors, prohormone convertases, granins, and other DCG proteins are secreted through the regulated or constitutive secretory pathways. Even relatively subtle changes in protein processing, levels, or regulated secretion, due to specific SNPs that impact a variety of proteins in the secretory pathway, have been reported to be associated with neuropsychiatric or metabolic disease, and these studies are reviewed below.

### Neurotrophins

#### Brain-derived neurotrophic factor

Brain-derived neurotrophic factor (BDNF) is well known for its role in regulating neuroplasticity and neurogenesis in the brain. A functional variant in the propeptide domain of BDNF, SNP rs6265 (G- to A-allele, Val66Met), was found to affect the secretion of BDNF (14). Previous biochemical studies have shown that BDNF (Met66, encoded by minor A-allele) fails to bind to sortilin through its pro-domain region, and this lowers its activity-dependent secretion due to failed DCG sorting and localization (14, 15). BDNF (Met66) is associated with reduced brain volume, impaired episodic memory (16), and high trait anxiety (17). Two other SNPs located at the protease cleavage site of the proBDNF protein, SNPs rs1048220 (G- to T-allele, Arg125Met) and rs1048221 (G- to T-allele, Arg127Leu), partially impair proBDNF cleavage but not DCG sorting and secretion (18). The minor alleles of SNP rs1048220 (T-allele) and SNP rs1048218 (T-allele) have also been associated with familial and sporadic Alzheimer’s disease (19).

#### Nerve growth factor

Mature nerve growth factor (NGF) binds to tropomyosin-related kinase A (TrkA) receptor tyrosine kinase and activates signaling pathways that regulate neuronal differentiation and survival. Unprocessed proNGF, however, has higher binding affinity for the p75NTR neurotrophin receptor, and can stimulate either cell survival or programed cell death (20). SNP rs6330 (C- to T-allele, Ala35Val) was previously identified in the pro-domain region of NGF, which has the potential to alter the efficiency of proNGF sorting or processing. The nucleotide variant of SNP rs6330 was found to be associated with increased anxiety in women (C-allele) (21), was over-transmitted in Attention Deficit Hyperactivity Disorder (ADHD)-affected children (C-allele) (22), and was associated with Alzheimer’s disease onset (both C- and minor T-alleles) (19, 23).

### Neuropeptides

#### Proopiomelanocortin

Expression of proopiomelanocortin (POMC), the precursor of several processed neuropeptides [α-MSH, adrenocorticotrophic hormone (ACTH), β-MSH, and β-endorphin], is regulated by the adipocyte-derived hormone leptin, which signals by binding receptors on neurons in the hypothalamic arcuate nucleus. The POMC-derived peptide α-MSH activates the melanocortin 4-receptor (MC4R) and suppresses food intake. Lack of POMC in humans and mouse models leads to the development of severe obesity, ACTH deficiency, and hypopigmentation (24, 25). A missense amino acid substitution by SNP rs28932472 (G- to C-allele, Arg236Gly) in the *POMC* gene was reported to disrupt the dibasic processing site between β-MSH and β-endorphin. The minor C-allele that encodes POMC (Gly236) results in an aberrant fusion peptide of β-MSH and β-endorphin, which still binds to the MC4R but antagonizes its activation. As a consequence, the Gly236 variation is associated with early onset obesity in several ethnic groups (26).

#### Agouti-related peptide

Expressed in the arcuate nucleus of the hypothalamus, agouti-related peptide (AgRP) is an endogenous antagonist of the MC4R that increases feeding behavior. The amino acid substitution of Ala67 to Thr67 caused by SNP rs5030980 (G- to A-allele) in the *AgRP* gene is associated with Anorexia Nervosa and leanness (27). In healthy subjects, homozygosity for the Thr67 allele is associated with low body fat mass and low lean body mass, while the Ala67 allele is associated with late-onset obesity. Although the Thr67 variation results in no detectable change in binding to the MC4R or sorting efficiency into and secretion from DCGs, the polarity of the amino acid substitution may cause a conformational change in AgRP, affecting other peptide functions that need further characterization (28).

#### Neuropeptide Y

Neuropeptide Y (NPY) is a potent orexigenic peptide that is also expressed in the hypothalamus. It regulates energy balance through effects on energy intake, expenditure, and partitioning. A non-synonymous SNP rs16139 that leads to an amino acid change (T- to C-allele, Leu7Pro) in the signal peptide domain of preproNPY has been reported to cause a tertiary structural change in its sorting domain, and this Pro7 substitution alters intracellular proNPY packaging, processing, and secretion (29–31). NPY (Pro7) is associated with elevated food intake, altered free fatty acid (FFA) metabolism and high serum cholesterol and LDL cholesterol levels, but doesn’t affect insulin sensitivity, insulin secretion, or glucose metabolism (30, 32, 33). Lower plasma NPY and norepinephrine concentrations, and lower insulin but higher glucose concentrations in plasma, were also reported in the population with the Pro7 substitution (34).

### Hormones

#### Insulin

A nucleotide variation in the proinsulin gene is located at the C-peptide-A-chain junction (C65, causes Arg to His), and the His65 substitution prevents processing of the dibasic cleavage site, resulting in hyperproinsulinemia that is caused by the accumulation of a circulating, biologically defective form of the proinsulin intermediate peptide, which fails to be metabolized via receptor-mediated endocytosis (35). The other identified proinsulin nucleotide variation results in an amino acid substitution of B10 (His to Asp), resulting in aberrant proinsulin sorting into the constitutive secretory pathway and a subsequent failure in peptide processing, which is also associated with hyperproinsulinemia in affected individuals (36, 37).

### Granins

Proteins of the granin family, including the chromogranins and secretogranins, have been demonstrated to play an important role in DCG biogenesis, in neural, neuroendocrine, and endocrine cells (2). It is not too surprising, then, that a number of SNPs which alter granin expression levels have been associated with metabolic diseases or neurological disorders, because physiological homeostasis is tightly regulated by neuropeptides, growth factors, and hormones, all of which are processed and stored in DCGs.

#### Chromogranin A (CHGA)

The combination of SNPs that are inherited together is called a haplotype, which can be used for studying genetic linkage of diseases. Two haplotype polymorphism carriers, haplotype (A-T-C) of SNPs rs9658634–rs9658635–rs7159323 in the *CHGA* promoter region, and haplotype (T-C) of SNPs rs7610–rs875395 in the *CHGA* 3′UTR and downstream regions, are linked to hypertensive renal disease (38). SNP rs9658634 in the *CHGA* promoter was found to be located in a predicted PPARγ/RXRα binding motif, and the nucleotide variant A-allele disrupted reporter expression that was co-stimulated by PPARγ/RXRα and their cognate ligands. Physiologically, the minor A-allele is associated with lower leptin secretion, as well as lower BMI, especially in women (39). SNP rs7610 (minor T-allele) has been identified in *CHGA*, showed decreased reporter expression in PC12 pheochromocytoma cells, and was associated with lower plasma chromogranin A levels and blood pressure (BP) in a sex-dependent manner (40). Other polymorphisms identified in *CHGA* coding sequence (rs9658667, G- to A-allele, Gly364Ser, and rs9658668, C- to T-allele, Pro370Leu) result in altered catestatin activity, changing its potency to inhibit nicotinic acetylcholine receptor (nAChR)-stimulated catecholamine release from chromaffin cells, and likely linking these SNPs to an increased risk of developing hypertension (41, 42). SNP rs9658635 and haplotype (C-T) of SNPs rs9658635–rs729940 are both linked to the onset of schizophrenia in the Japanese population, but their effect on *CHGA* gene expression remains to be determined (43).

#### Chromogranin B (CHGB)

Reduced chromogranin B levels have been observed in the thalamic subregion, the mediodorsal nucleus, parvocellular division (MDNp; *CHGB* mRNA levels), and cerebrospinal fluid (both CgA and CgB protein levels) of schizophrenic patients (44, 45). Two polymorphisms identified in the *CHGB* coding sequence, rs236152 (C- to G-allele, Arg353Gly) and rs236153 (A- to G-allele, Glu368Glu), are associated with schizophrenia in a Japanese population study, although the functional consequences of these two SNP variants are unknown (46). A haplotype (A-T) of SNPs rs236140–rs236141 identified in the *CHGB* gene promoter region shows the highest transcriptional strength in reporter assays carried out in PC12 cells, and interestingly, the (A-T) haplotype is strongly associated with hypertension (47). The SNP rs2821 (minor A-allele) found in an RNA-destabilizing A/U-rich motif of the *CHGB* 3′UTR was reportedly associated with lower plasma chromogranin B levels in population studies, likely due to shortened *CHGB* mRNA half-life (48).

#### Secretogranin II and secretogranin III (SCG2 and SCG3)

Single nucleotide polymorphism rs1017448 (minor A-allele) was found in the first intron of the *SCG2* gene. It is significantly associated with elevated BP, and likely increases *SCG2* gene expression by facilitating the recruitment of paired-like homeobox transcriptional factor 2a (Phox2a) (49). SNP rs3764220 (A-allele), which is found in the promoter region of the *SCG3* gene, is associated with metabolic syndrome-related physiological changes, including dyslipidemia, hypertension, and impaired glucose tolerance, with increased risk of cardiovascular disease-related morbidity and mortality (50). SNP rs16964465 (minor C-allele) in the *SCG3* promoter region and minor G-allele of rs16964476 in the first intron, both enhanced transcriptional activity of a reporter in the neuroblastoma cell line SH-SY5Y (51). Interestingly, in hypothalamus, SgIII protein is detected in both POMC and NPY neurons in the arcuate nucleus, and in orexin-expressing and melanin-concentrating hormone (MCH)-expressing neurons in the lateral hypothalamus; all these neuropeptides regulate food intake (51). Notably, these two minor alleles, both associated with increased SgIII expression, seemed to confer resistance to the onset of obesity, suggesting the possibility that decreased SgIII levels could reciprocally increase the risk of obesity.

### Peptidases and convertases

#### Prohormone convertase 1/3

Prohormone convertases are a family of endopeptidases that cleave proteins at internal sites, generally paired or clustered basic lysine, and/or arginine residues. PC1 is encoded by the proprotein convertase, subtilisin/kexin-type 1 (*PCSK1*) gene. Two functionally relevant SNPs, originally identified in different *PCSK1* alleles of a female patient with childhood early onset obesity, are associated with abnormal glucose homeostasis, and elevated plasma proinsulin and POMC levels (52, 53). SNP rs137852821 (G- to A-allele) changes an amino acid from Gly483 to Arg483, which prevents processing of proPC1 and favors its retention in the RER. Another C-allele variant found in the intron-5 donor splice site of *PCSK1* gene also causes exon 5 skipping and results in premature termination of PC1 translation in the catalytic region. Yet another identified non-synonymous allele substitution that is associated with early onset obesity is SNP rs6232 (A- to G-allele), which changes Asn221 to Asp221 and causes impaired catalytic function of PC1 (54).

#### Carboxypeptidase E

Carboxypeptidase E trims C-terminal lysine and arginine residues from peptides that are generated by prohormone convertase cleavage of precursor proteins at paired dibasic residues (55). Notably, CPE is also a sorting/retention receptor for proinsulin and proBDNF trafficking to the regulated secretory pathway (56, 57). The SNP rs144727363 (C- to T-allele, Arg189Trp) in the *CPE* coding region changes arginine to tryptophan, which reduces enzymatic activity of CPE, and is associated with hyperproinsulinemia and type II diabetes mellitus in affected Ashkenazi Jewish families (58). Similarly, a missense mutation in the *CPE* gene (Ser202Pro), identified from the mouse *fat/fat* model, which generates an enzymatically inactive and unprocessed protein product, results in impaired processing of proinsulin and other propeptides, chronic hyperproinsulinaemia, and obesity (59–61).

### Other proteins that regulate secretory pathway function

#### ATP6V0A1

*ATP6V0A1* encodes the α1 subunit of the vacuolar H^+^-translocating ATPase complex, which functions in acidification of intracellular organelles. Bafilomycin A1, a chemical inhibitor of the vacuolar H^+^ ATPase, impairs DCG formation, and the sorting of secretory proteins into the regulated pathway (62). A reported SNP rs938671 (minor C-allele, 3246 T/C) in the 3′UTR of *ATP6V0A1* gene creates a binding motif for the micro-RNA hsa-miR-637, which decreases overall gene expression of *ATP6V0A1*. Its clinical association with hypertension may be due to altered DCG acidification by the risk C-allele, and as a consequence, decreased sorting, retention, and/or processing of DCG components (63).

#### Sortilin (SORT1)

Sortilin, a Vps10p domain protein, binds to proBDNF, and other polypeptides and facilitates their trafficking into the regulated secretory pathway (15). Plasma membrane sortilin also functions as an internalization receptor for apolipoprotein A-V and progranulin uptake (64, 65). Several SNPs are located in a non-coding region downstream of the *SORT1* allele and strongly correlate with increased sortilin expression, including SNP rs12740374 (minor T-allele), which creates a C/EBPα binding site, SNP rs646776 (minor C-allele), and SNP rs599839 (minor G-allele). Increased sortilin expression is reported to associate with reduced ApoB secretion, enhanced LDL uptake in the liver, and increased uptake of plasma progranulin (66–69). How these existing SNPs affect other known function of sortilin, including propeptide sorting, is still unclear.

## Future Perspectives

Protein secretion from neuroendocrine, neural, and endocrine cells is a complex, highly regulated process. Due to the nature of the cargo, which includes critical growth factors, hormones, peptide precursors, the enzymes that process them, and proteins that function in secretory vesicle biogenesis, subtle alterations in the regulated secretory pathway, and/or the proteins transiting through it, can have a significant physiological impact. Many studies reviewed here describe the association of specific, discrete SNPs in the genes of secreted proteins with the risk or onset of human disease. Neuropsychiatric disorders such as schizophrenia, metabolic disorders including obesity and diabetes, and hypertension are strongly associated with a number of well-characterized SNPs. These identified SNPs together with genome-wide association studies (GWAS) and molecular and cellular analyses of SNP function will advance our understanding of the process of regulated secretion and the important roles that secreted and secretory proteins play in maintaining physiological homeostasis.

## Conflict of Interest Statement

The authors declare that the research was conducted in the absence of any commercial or financial relationships that could be construed as a potential conflict of interest.
